# Book Reviews and Notices

**Published:** 1888-07

**Authors:** 


### BOOK REVIEWS AND NOTICES.

THE LANGUAGE OF MEDICINE. A manual giving the origin, etymology, pronunciation, and meaning of the technical terms found in medical literature. By F. R. CAMPBELL, A. M., M. D. Pp. viii and 318. New York: D. Appleton & Co. 1888. Chicago: A. C. McClurg & Co.

The author says that "the object of this work is to provide the medical student with a suitable means of acquiring the vocabulary of his science." This is a commendable object, and both the book and the subject deserve more attention than the latter has received during recent years. The author claims that too little attention is given in medical education to making students men of general and linguistic culture, that the tendency of the profession is away from those lines which made the physicians of former generations scholarly men rather than scholars, and he deplores the fact. He says that too many physicians abbreviate the Latin of their prescriptions simply to conceal their ignorance.

Part I, treats of the origin of the language of medicine, *i. e.*, the words which are peculiar to medical language. Part II, considers the Latin element in the language. Part III, the Greek, and Part IV, the elements derived from modern languages.

It is not claimed that the book is a complete medical lexicon, and there has been no attempt to make it such. The principal words, however, from both the Greek and Latin are given, together with prefixes, suffixes, prepositions, and clearly stated rules for their combination, inflection, and correct use in prescription writing. An ample number of examples are furnished for illustration, and exercises are also given for those who need them. The chapter containing a list of the most commonly mispronounced words will prove profitable reading for many doctors.

Those physicians who have the training of the colleges of this country, will find ample material in the book to verify the quotation with which this book notice begins. But whether those wholly untrained in Greek and Latin will fare so well can be determined only by a trial. Probably, however, they will.

The author's style is both good and attractive, and the publisher's work is exceptionally well done. The book will be found convenient for reference, suitable for a text-book, and profitable reading for all classes of medical practitioners. If, as the author suggests, the work should, perhaps, have been done by a professor of language, the medical profession is to be congratulated that he did not wait for any such desideratum. E. W.

MEDICAL PUBLICATIONS, HARVARD MEDICAL SCHOOL. J. COLLINS WARREN, chairman of the committee on publication. 1887.

There are some 200 pages in this book, which is composed of articles contributed by the medical corps of the school, and represent only a portion of the original work of the year 1887. The articles are reports of work in experimental anatomy, physiology, pathology, and surgery, and a consideratiou of the artificial feeding of infants. The papers are excellent, and represent very creditable work; but the great variety in their nature precludes special mention in this connection.

FOURTEENTH ANNUAL REPORT OF THE Secretary of the State Board of health of Michigan, for the fiscal year ending September, 30, 1886. Lansing, Mich.: Thorp & Godfrey. 1888.

The first part of this report, xlix pages, contains the official records of the meetings of the board. In the second part, of 324 pages, the papers which are of greatest interest to the profession are the now celebrated reports of Professor Vaughn, upon the presence of tyrotoxicon in poisonous ice cream; and that of Dr. Baker, secretary of the board, upon the causation of pneumonia. The work which the secretary is doing in observations and reports upon meteorological conditions, and upon the prevalence of communicable disease in Michigan is highly creditable.

A MANUAL OF PHYSIOLOGY. A TEXT-book for students of medicine. By GERALD F. YEO, M. D., F. R. C. S. Third American, from the second English, edition. Pp. xxi and 758. Philadelphia: P. Blakiston, Son & Co. 1888.

In the preparation of this edition the changes rendered necessary by recent research have been made, together with a correction of inaccuracies.

The favor with which this treatise has been received, shows very clearly how successful the author has been in his attempt to write a book for the use of medical students. No one can know what the relative value of the many facts of physiological science is to such students, as well as a practical medical man does. In the short time allotted by even our best colleges for the work it is impossible to make of the average medical student, both a good physician and a good physiologist. The author fully appreciates this fact, and it is along this line that the merit of the work is most conspicuous; but there are other merits in the volume. The style is clear and agreeable. The drawing of histological structures is exceptionally true, and those of Dr. Bevan Lewis of the spinal cord and brain of monkeys can only be described as beautiful in their delicacy and accuracy. E. W.

A COMPEND OF HUMAN PHYSIOLOGY. Especially adapted for the use of medical students. By ALBERT P. BRUBAKER, A. M., M. D. Fourth edition, revised and enlarged. Pp. viii and 174. Philadelphia: P. Blakiston, Son & Co. 1888.

This is No. 4, in the "Quiz Compend" series issued by its publishers. It is especially intended for the use of students preparing for examination. For this purpose it is difficult to say in what manner this compend can be improved. It deserves its popularity.

THE RECTUM AND ANUS. THEIR DISEASES and Treatment. By CHARLES B. BALL, F. R. C. S. I., Dublin. With 54 illustrations, and colored plates. 12mo. Pp. 410. Philadelphia: Lea Brothers & Co. Chicago: A. C. McClurg & Co. 1888.

Of the numerous manuals lately written upon this subject, this book is one of the best.

Each subject is concisely and systematically treated; its literature well presented and deductions carefully drawn. The chapter upon malformations is comprehensive, and is interesting reading.

The author's descriptions of operations aided by drawings are easily understood, though the relative advantage of one method of procedure over another for the relief of the same condition, is not greatly emphasized.

The lithographic plates show rather too much color to be faithful representations. H. H. F.

A REFERENCE HANDBOOK OF THE MEDICAL Sciences. Embracing the entire range of scientific and practical medicine and allied science, by various writers. Vol. VI, Pra to Tep. 778 pages. Illustrated by chromo-lithographs and wood engravings. Edited by ALBERT H. BUCK, M. D. Supplied to subscribers only. New York: William Wood & Co. Chicago: W. T. Keener.

This sixth volume of the *Reference Handbook* sustains the character of the five volumes which have preceded it. It is comprehensive in its scope, and the subjects are well presented.

It is printed with clear type on good paper.

When completed the set will constitute a small library in itself.

THE TREATMENT OF HÆMORRHOIDS BY injections of carbolic acid and other substances. By SILAS T. YOUNT, M. D. Second edition. Duodecimo, pp. 102. Echo Music Co., La Fayette, Indiana. 1888.

A valid excuse for the existence of this volume is hardly apparent, as it contains nothing at all new (except the description of a speculum invented by the author), and the book is too small to treat thoroughly the subjects upon which it touches. The work abounds in grammatical errors.

SURGERY—ITS THEORY AND PRACTICE. By WM. JOHNSON WALSHAM, F. R. C. S. With 236 illustrations. 8vo. Pp. ix and 655. Philadelphia: Blakiston, Son & Co. Chicago: W. T. Keener. 1887.

Individual points of excellence are likely to be few as distinguishing any one *Manual of Surgery*—intended for students' use—from another on the same subject, as such works are almost of necessity compiled and extracted from other and more extensive treatises; the chief value of the manual being that it presents the subject in condensed form.

The volume under consideration is more nearly complete than most of its kind; is systematic and clearly written. The illustrations are numerous, some of them new, and a few of them good.

H. H. F.

ACCIDENTS AND EMERGENCIES. A Manual of the treatment of surgical and other injuries, in thé absence of a physician. By CHARLES W. DULLES, M. D. Third edition, illustrated. Duodecimo. Pp. viii and 119. Philadelphia: P. Blakiston, Son & Co. Chicago: W. T. Keener.

This little book is full of practical suggestions, so worded as to be easily understood and acted upon by any person of average common sense and discretion in a way likely to benefit the patient, and in many cases to save life.

The volume should find a place in every household, especially if situated at a distance from medical aid. The fact that it has reached its third edition is in itself a recommendation. H. H. F.

